# Exploring alternative insecticide delivery options in a “lethal house lure” for malaria vector control

**DOI:** 10.1038/s41598-023-31116-7

**Published:** 2023-03-24

**Authors:** Welbeck A. Oumbouke, Antoine M. G. Barreaux, Innocent T. Zran, Alphonsine A. Koffi, Yao N’Guessan, Ludovic P. Ahoua Alou, Rosine Z. Wolie, Jackie Cook, Eleanore D. Sternberg, Matthew B. Thomas, Raphael N’Guessan

**Affiliations:** 1 Department of Disease Control, London School of Hygiene and Tropical Medicine, London, UK; 2 Innovative Vector Control Consortium, IVCC, Liverpool, UK; 3 Institut Pierre Richet (IPR)/Institut National de Santé Publique (INSP), Bouaké, Côte d’Ivoire; 4 Department of Entomology, Center for Infectious Disease Dynamics, The Pennsylvania State University, University Park, PA 16802 USA; 5 Animal Health Theme, ICIPE, Nairobi, Kenya; 6 CIRAD, UMR INTERTRYP, 34398 Montpellier, France; 7 CIRAD, IRD, INTERTRYP, Univ Montpellier, 34000 Montpellier, France; 8 Unité de Recherche et de Pédagogie de Génétique, UFR Biosciences, Université Félix Houphouët-Boigny, Abidjan, Côte d’Ivoire; 9 Medical Research Council (MRC) International Statistics and Epidemiology Group, London School of Hygiene and Tropical Medicine, London, UK; 10 Tropical Health LLP, London, UK; 11 Entomology and Nematology Department, University of Florida, Gainesville, USA

**Keywords:** Medical research, Malaria

## Abstract

The In2Care EaveTube is a house modification designed to block and kill malaria mosquitoes using an electrostatic netting treated with insecticide powder. A previous study demonstrated prolonged duration of effective action of insecticide-treated electrostatic netting in a semi-field setting. As part of a cluster randomized controlled trial (CRT) of the EaveTube intervention in Côte d’Ivoire, we investigated the residual efficacy of a pyrethroid insecticide deployed in EaveTubes under village conditions of use. We also explored the scope of using existing malaria control technologies including LLINs and IRS as alternative methods to deliver insecticides in the lethal house lure. The efficacy of beta-cyfluthrin was monitored over time using the “eave tube bioassay” method. Mortality of beta-cyfluthrin exposed pyrethroid resistant *Anopheles gambiae* mosquitoes was > 80% after 4 months. The impact (mosquito mortality) of PVC tubes coated with pirimiphos methyl was similar to that of beta-cyfluthrin treated insert (66.8 vs. 62.8%) in release-recapture experiments in experimental huts. Efficacy was significantly lower with all the LLINs tested; however, the roof of PermaNet 3.0 induced significantly higher mosquito mortality (50.4%) compared to Olyset Plus (25.9%) and Interceptor G2 (21.6%) LLINs. The efficacy of the alternative delivery methods was short-lived with mortality decreasing below 50% within 2 months in residual activity bioassays. None of the products tested appeared superior to the powder treatments. Further research is therefore required to identify suitable insecticide delivery options in EaveTube for malaria vector control.

## Introduction

The primary methods of malaria vector control currently in use are long lasting insecticidal nets (LLINs) and indoor residual spraying (IRS). These methods prevent disease transmission by targeting mosquito behaviours that occur inside of houses, namely blood feeding and resting^1,2^. Even though these strategies have contributed to most of the recent reduction in malaria burden across sub-Saharan Africa^3^, the disease remains an important public health problem, claiming about half a million lives annually^4^. New tools that target mosquitoes surviving exposure to insecticide treated surfaces^5^ and those biting outside of sleeping hours and outdoors^6^ are required to build on the recent gains, and meet the control target set forth in the World Health Organization (WHO) Global Technical Strategy^7^.

An improved understanding of mosquito ecology and behaviour^8^ could inform the design of new strategies of control. There is evidence that African malaria vectors have a strong preference for using eave gaps (the space between the roof and the wall) found in many traditional African houses as an entry point. This behaviour offers vector control opportunities; for example host-seeking mosquitoes could be prevented from entering houses through the blocking of eave gaps and other openings in house walls^9,10^. Evidence from a number of observational and randomized controlled trials suggest that house modifications which prevents mosquito entry is associated with reduction of indoor mosquito biting and transmission of malaria^11–14^. Although house improvement has contributed to malaria elimination in developed countries, its potential as a vector control tool remains largely underexploited in Africa. However, there is increasing interest in adding house improvement to the current malaria control arsenal^15^.

While blocking eaves of houses prevent mosquito entry, the strong affinity that mosquitoes have for this opening means that it can be targeted for insecticide treatment. In2care EaveTube is a house modification intervention classified generically as a “lethal house lure” (https://apps.who.int/iris/bitstream/handle/10665/274451/WHO-CDS-VCAG-2018.03-eng.pdf) by the WHO Vector Control Advisory Group (VCAG). The EaveTube intervention consists of taking sections of plastic pipe and fitting them with a screened insert and installing them into a closed eave space. The electrostatic netting insert placed inside the tube is treated with an insecticide powder formulation that delivers a lethal dose to mosquitoes as they attempt to enter houses to blood feed. Thus, the lethal house lure, in this case, consists of a physical component comprised of netting insert (blocking mosquito entry) and a chemical component (insecticide) used to treat the netting. The potential for EaveTubes, combined with general house improvement to block entry of mosquitoes (e.g. filling gaps in the eaves, screening windows, repairing doors etc.) to control malaria vectors and reduce transmission was demonstrated in a number of semi-field and modelling studies^12,13,16–18^. Further, a recent cluster randomized controlled trial demonstrated a 38% reduction in malaria incidence in children living in houses fitted with In2Care EaveTubes plus house screening, on top of standard pyrethroid LLIN, in a high malaria transmission and pyrethroid resistance area in central Côte d’Ivoire^19^.

The insert inside the In2Care EaveTube has a special electrostatic coating which enhances the bioavailability of powder formulated insecticides on the netting surface^20^. Evidence from previous work shows that various active ingredients and formulations can be deployed on electrostatic netting to good effect when freshly applied^20^, but only the pyrethroid beta-cyfluthrin was effective over an extended period (9 months)^16^, although this measure of residual activity was obtained under controlled conditions.

While electrostatic netting treated with insecticide holds potential for controlling insecticide resistant mosquitoes, there is scope for tapping into alternative insecticide delivery technologies including new generation LLINs and IRS insecticides to achieve a similar effect when inserted or applied in an eave tube-like delivery system^21^. New LLINs are coming to market, treated with a mixture of a pyrethroid and either a synergist (piperonyl butoxide (PBO)^22–25^), an insect growth regulator (pyriproxyfen (PPF)^26–28^) or a pyrrole insecticide (chlorfenapyr^29–32^). Similarly, there are new IRS products formulated with the organophosphate insecticide pirimiphos methyl^33^, the neonicotinoid clothianidin^34^ or the meta-diamide broflanilide^35^. These new products are effective against pyrethroid resistant mosquitoes and so in principle, could be deployed as a lethal house lure in areas with pyrethroid resistant vectors. The capacity for production and deployment of IRS and LLIN-type products is also well established and so leveraging these technologies could facilitate wide-scale implementation of the approach.

The present study aimed to investigate: (1) the residual activity of pyrethroid treated In2Care EaveTube inserts under field conditions, and (2) proof of principle for alternative ways of delivering insecticides in a lethal house lure, either by using netting from new generation LLINs or dipping the tube in insecticide solutions.

## Methods

### Mosquitoes

Experiments were conducted with *Anopheles gambiae* sensu lato (s.l.) mosquitoes collected around Bouaké, central Côte d’Ivoire. This mosquito population has a high prevalence of resistance to the major classes of mosquito adulticides, including pyrethroids^36–38^. Mosquitoes were collected as larvae from breeding sites using the dipping method and reared to adult in insectary under controlled temperature and humidity conditions (27 ± 2 °C, 60 ± 20% RH). Larvae were fed on ground Tetramin baby fish food. Adult mosquitoes emerging from pupae were placed in 30 cm x 30 cm netted cages and maintained on 10% honey solution until testing.

### Residual activity of beta-cyfluthrin treated EaveTube inserts under field conditions

This assessment was done as part of a cluster randomised controlled trial (CRT) in central Côte d’Ivoire. Forty villages were selected for the CRT with half assigned to household screening plus EaveTubes (SET) and the other half as controls^39^. All villages received new LLINs, so the aim of the CRT was to investigate whether SET provides added protective benefit against malaria transmission on top of LLINs. Beta-cyfluthrin was selected for the CRT because this product was registered for use in country and results from a previous study indicated a long-lasting activity (> 9 months) of this pyrethroid on electrostatic EaveTube inserts under controlled, semi-field conditions^16^.

Inserts fitted to houses in the 20 intervention villages were machine-treated by In2care with an undiluted wettable powder formulation of 10% beta-cyfluthrin (Tempo 10, Bayer). The dose of insecticide applied was in the range 300–500 mg per insert.

To monitor the efficacy of treated inserts under field conditions in real houses, residual activity was tested monthly using a subsample of inserts from study villages using the eave tube bioassay method.

The procedure of this bioassay was described in detail in Oumbouke et al.^16^. In brief, the assay comprises of a 20-cm long plastic tube containing an insert such that it is flush with one end of the tube and mosquitoes are introduced into the tube through the opposite end, which is fitted with an untreated netting to keep mosquitoes inside the tube. A 1.5L plastic bottle filled with hot water and wrapped in socks worn the previous night was placed behind the insert and served as a host cue. Mosquitoes attracted to the heat and odour cues then contact the insecticide-laden insert. The eave tube assay is similar to the previously described MCD bottle assay^40^ in that both mimic the interaction between host-seeking mosquitoes and insecticide-treated surfaces. To increase host-seeking activity, mosquitoes were sugar starved for 6 h prior to testing. Approximately 100 mosquitoes in batches of 20–25 were exposed for 1 h in the eave tube bioassay. Following exposure, mosquitoes were released in netted cages and provided with 10% honey solution and mortality scored after 24 h.

Four beta-cyfluthrin treated inserts were sampled from each EaveTube village every month for testing. The number of inserts tested was based on logistical constraints in the field. Bioefficacy testing was performed monthly until activity decreased below 80% mortality at which point all the inserts in the villages were replaced with freshly treated inserts.

### Semi-field evaluation of two alternative insecticide delivery approaches in EaveTubes

#### Insecticide treatments

**Figure 1:**
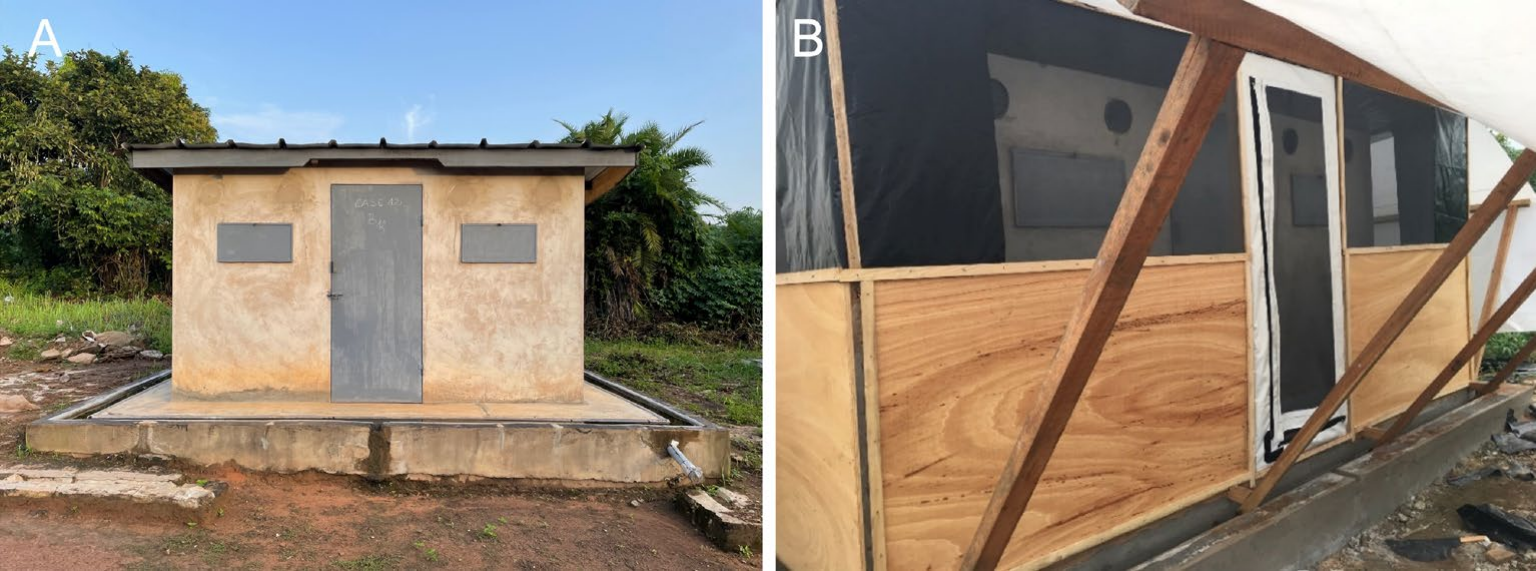
(**A**) West African experimental hut style at the M’Be rice field, central Côte d’Ivoire, (**B**) West African experimental hut fitted with eave tubes and surrounded with an enclosure.

Insecticide-treated electrostatic netting in tubes was shown to produce a significant reduction in overnight mosquito survival in previous semi-field studies^12,16–18^. The experiments described here explore alternatives to electrostatic netting for delivering insecticides in this system. The following delivery methods were tested in experimental huts surrounded by enclosure (Fig. 1) at the M’bé field station near Bouaké, central Côte d’Ivoire:

In2Care EaveTube inserts (In2Care, the Netherlands) coated with an undiluted wettable powder formulation of 10% beta-cyfluthrin (Tempo 10, Bayer) serves as a positive control. The dose of insecticide applied was in the range 300–500 mg per insert.

PermaNet 3.0 is a long-lasting insecticidal net manufactured by Vestergaard S.A. (Switzerland). The top panel, which was tested in the present study, is made of monofilament polyethylene (100 denier) fabric and treated with a mixture of the pyrethroid deltamethrin at 4 g/kg and the synergist piperonyl butoxide (PBO) at 25 g/kg. The side panels (not tested here) are made of multi-filament polyester (75 denier) fabric with a strengthened lower part incorporated with deltamethrin at 2.8 g/kg.

Olyset Plus is a long-lasting insecticidal net manufactured by Sumitomo Chemical (Japan). The net is made of 150 denier high-density mono-filament polyethylene yarn incorporating a mixture of the pyrethroid permethrin at 20 g/kg and PBO at 10 g/kg on all net panels.

Interceptor G2 is a long-lasting net manufactured by BASF (Germany). The net is a dual-active LLIN made up of knitted multi-filament polyester fibres incorporating a mixture of the pyrethroid alpha-cypermethrin at 2.4 g/kg and the pyrrole insecticide chlorfenapyr at 4.8 g/kg.

The organophosphate pirimiphos methyl is a WHO recommended insecticide used extensively in IRS campaigns. Capsule suspension formulation of pirimiphos methyl (Actellic CS, Basel, Switzerland) was tested in the present study.

### Semi-field enclosure

Semi-field performance of the alternative tube treatments was tested in two experimental huts at the M’bé field station, near Bouaké, central Côte d’Ivoire. The huts are of the West African design^41^, 3.25 m long, 1.76 m wide and 2 m high. The interior walls of the huts are made of concrete brick, with a corrugated iron roof. A plastic cover was affixed onto the roofing as ceiling. Each hut was built on a concrete base with a water-filled moat, to prevent invertebrate predators from preying on dead or knocked down mosquitoes. A number of modifications were made to the huts for these experiments: (1) six holes were drilled at eave level (1.7 m from the ground) on three sides of the hut (two holes on each side), (2) insecticide treated tubes were fitted into the holes, (3) an enclosure was built around each hut to allow recapture of mosquitoes outside of the hut (Fig. 1). The semi-field enclosure consists of a wooden frame erected on the concrete base, 50 cm from the exterior wall of the hut. The roof was made of plastic sheeting which extended beyond the edge of the enclosure as an overhang to prevent rain from entering. The bottom half of the frame was made from wooden panels and the top half was screened with polyethylene netting. White plastic sheeting was installed on the floor of the enclosure to facilitate the collection of dead mosquitoes. A zipper access door was positioned on the front side of the hut to allow entry into and exit from the enclosure.

#### Release-recapture experiments

In the first experiment, six 30 cm × 30 cm netting samples were cut from the LLINs and fitted in tubes in one experimental hut (the intervention). Six pieces of untreated netting of the same size were placed in the second experimental hut, located 50 m away (the control). The netting samples were cut from Olyset Plus and Interceptor G2 and from the roof panel of PermaNet 3.0 and evaluated on different occasions.

In a second experiment, tubes were dipped in aqueous solution of pirimiphos methyl at 10 g/m^2^. The tubes were treated by rolling one tube at a time in insecticide solution for 5 min and subsequently left to dry for 24 h before testing. Tubes treated with pirimiphos methyl were screened with untreated netting (the intervention). A control hut fitted with untreated tube containing untreated netting was tested in parallel (the control). In the third experiment, six inserts freshly treated with beta-cyfluthrin were installed in one experimental hut (the intervention) and six untreated inserts were placed in tubes in a second experimental house (the control).

Two adult volunteers were recruited to sleep in the huts. Volunteer sleepers rotated between huts on consecutive nights to account for any potential difference in attractiveness to mosquitoes. The volunteers entered the hut at 20:00 h and slept under intact untreated nets. Approximately 100 non-bloodfed sugar starved 5-day old female *An. gambiae* mosquitoes were released into each enclosure every release night 15 min after sleepers entered their respective huts. Mosquitoes were recaptured the following day at 05:00 inside the enclosure. Mosquitoes collected were brought back to the laboratory at Institut Pierre Richet (IPR) in Bouake, Côte d’Ivoire for scoring of immediate mortality. Surviving mosquitoes were provided with 10% honey solution and any delayed mortality was scored up to 72 h later.

#### Sample size calculations

Evidence from previous semi-field studies suggests that insecticidal tube produces about 50% reduction in overnight mosquito survival^12,13,16,17^. Based on this, the number of release night required to detect a 50% reduction in survival with 80% power and significance level of 5% was determined for each treatment in the R software using the “pwr” package. Eight replicates of release-recapture were performed for each treatment, which according to the sample size calculation was above the number required to demonstrate the expected effect size.

### Insecticide susceptibility assays

Insecticide susceptibility assays were performed to measure susceptibility to the constituent actives in the LLINs and pirimiphos methyl in the local *An. gambiae* mosquito population. Discriminating concentrations of the pyrethroids deltamethrin (0.05%), permethrin (0.75%), alpha-cypermethrin (0.05%) and pirimiphos methyl (0.25%) were tested in WHO cylinders following WHO guidelines. A higher concentration of pirimiphos methyl (1%) was also tested in assays. Synergist assays were conducted by pre-exposing mosquitoes to PBO, which neutralises the activity of the cytochrome P450s involved in pyrethroid metabolism in mosquitoes. Because of stability issues with chlorfenapyr on filter paper, adapted Centre of Disease and Control (CDC) bottle bioassays were used to measure resistance to chlorfenapyr. Bottles were coated with chlorfenapyr at the discriminating dose of 50 µg/mL^42^. Four replicates of 25 female mosquitoes (sugar fed, aged 2–3 days) were exposed for 1 h to insecticide treated papers or bottles. Mortality was recorded 24 h (pyrethroids) and 72 h (chlorfenapyr) post-exposure. Mosquitoes in the control batch were held for 72 h before scoring mortality.

### Residual activity of new generation LLINs and pirimiphos methyl treatment

The residual activity of the best performing alternative delivery methods (PermaNet 3.0 roof and pirimiphos methyl coated PVC tube) in the release-recapture experiments was assessed.

Four 30 cm x 30 cm pieces from PermaNet 3.0 netting and four PVC tubes treated with pirimiphos methyl at the dosages of 1 g/m^2^ and 10 g/m^2^ were tested using the previously described eave tube assays^16^. Testing was performed on the netting pieces and the treated tubes at monthly intervals. To evaluate AI decay under realistic conditions, the pieces of the LLINs (installed in tubes) and the IRS treated tubes were stored between testing in holes drilled at eave level in an experimental hut at the institute. Four replicates of 25 non-blood fed 6 h sugar-starved, 5-day old mosquitoes were tested for each bioassay. Intervention and control mosquitoes were monitored for up to 72 h before scoring post-exposure mortality.

When mortality decreased below 50%, the netting samples were washed once and re-tested in the eave tube bioassays. Net washing was conducted following WHO guidelines^43^. Briefly, the pieces were washed individually for 10 min in a soap solution (savon de Marseille at 2 g/L of deionised water) using a shaker bath set at 155 movements/min and 30 °C. Samples were then rinsed twice in clean water for 10 min and left to dry for 3–4 h. Washed netting samples were tested only after full regeneration of the active ingredient (1 day)^44^.

### Chemical analysis

Content of deltamethrin and piperonyl butoxide was determined in the roof panel of unwashed PermaNet 3.0 netting at month 0, and the washed samples at month 2. Extraction of deltamethrin and PBO was performed using the CIPAC method^45^. Both compounds were extracted by refluxing with xylene for 30 min in the presence of dioctyl phthalate as internal standard and citric acid. The concentrations of deltamethrin and PBO were subsequently measured by Gas Chromatography with Flame Ionization Detection (GC-FID).

### Data analysis

All statistical analysis was performed using the R software version 3.5.3. Residual efficacy data across treatments was analysed using generalized linear models (GLMs) with the “arm” package. The models included insecticide treatments as independent variable and mosquito mortality as the outcome. Interactions between insecticides and residual efficacy testing interval were also included in the models. Pairwise comparisons were performed with the final model using the “multcomp” package. For the release-recapture experiments, generalized linear mixed models (GLMMs) with a binomial distribution and a logit link function was fitted to the data using the “lme4” package. The models included treatment as fixed effect. Enclosure, sleepers, and release-recapture study nights were treated as random effects. Significance of the fixed effect in the model was tested using likelihood ratio test (LRT). Susceptibility bioassay data were analysed using a χ 2-square test with Yates continuity correction.

### Ethical approval

Ethical clearance for the study was obtained from the ethics review committee of the London School of Hygiene and Tropical Medicine and the Côte d’Ivoire National Ethics Committee. Hut sleepers were all male and > 18 years old. Written informed consent was obtained from all volunteer sleepers taking part in the study prior to the release-recapture experiments. All experiments were carried out according to relevant national and international guidelines.

## Results

### Bioefficacy and residual activity of beta-cyfluthrin treated inserts deployed in study villages

**Figure 2:**
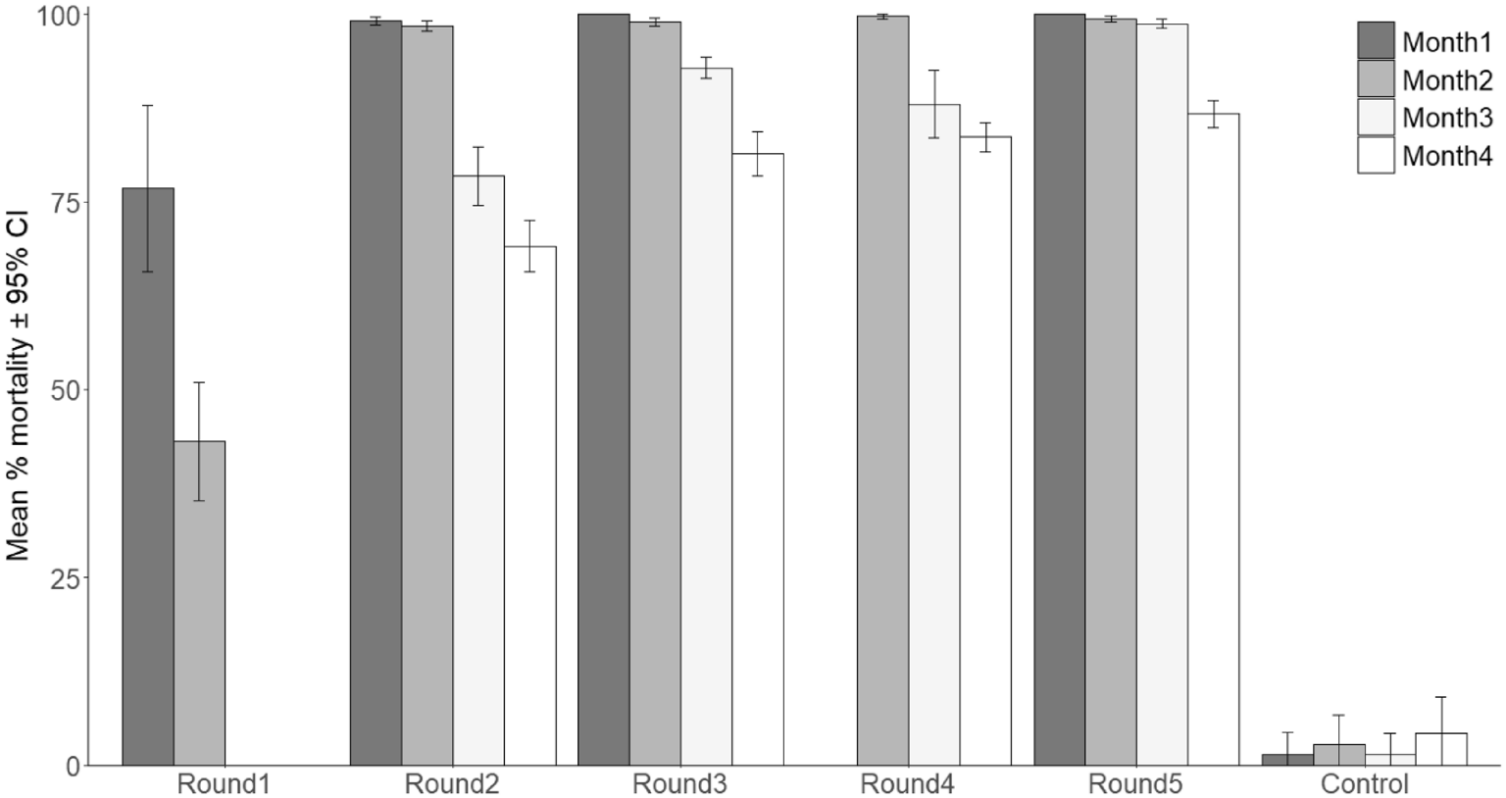
Average mortality of pyrethroid resistant *An. gambiae* mosquitoes exposed to beta-cyfluthrin treated inserts retrieved from trial villages. Bars represent average mortality for the 20 EaveTubes villages. Round indicates insert re-treatment cycle performed during the Eave Tubes trial; Round1 occurred between Mar 2017–May 2017, Round2: Jul 2017–Aug 2017; Round3: Dec 2017–Jan 2018; Round4: Apr 2018–May 2018 and Round5: Oct 2018–Nov 2018. Error bars indicate 95% confidence intervals.

The bioefficacy and the residual activity of beta-cyfluthrin treated inserts collected from study villages are presented in Fig. 2. Five rounds of insert retreatments were done over the two years of the cluster randomized controlled trial. Mortality of pyrethroid resistant mosquitoes exposed to the beta-cyfluthrin treated inserts from the first two treatment rounds was generally < 80% within only three months post-treatment (Fig. 2). However, beta-cyfluthrin appeared more durable in subsequent rounds, killing over 80% of the local pyrethroid resistant *An. gambiae* mosquitoes during the 4-month monitoring period.

### WHO susceptibility assays

**Figure 3:**
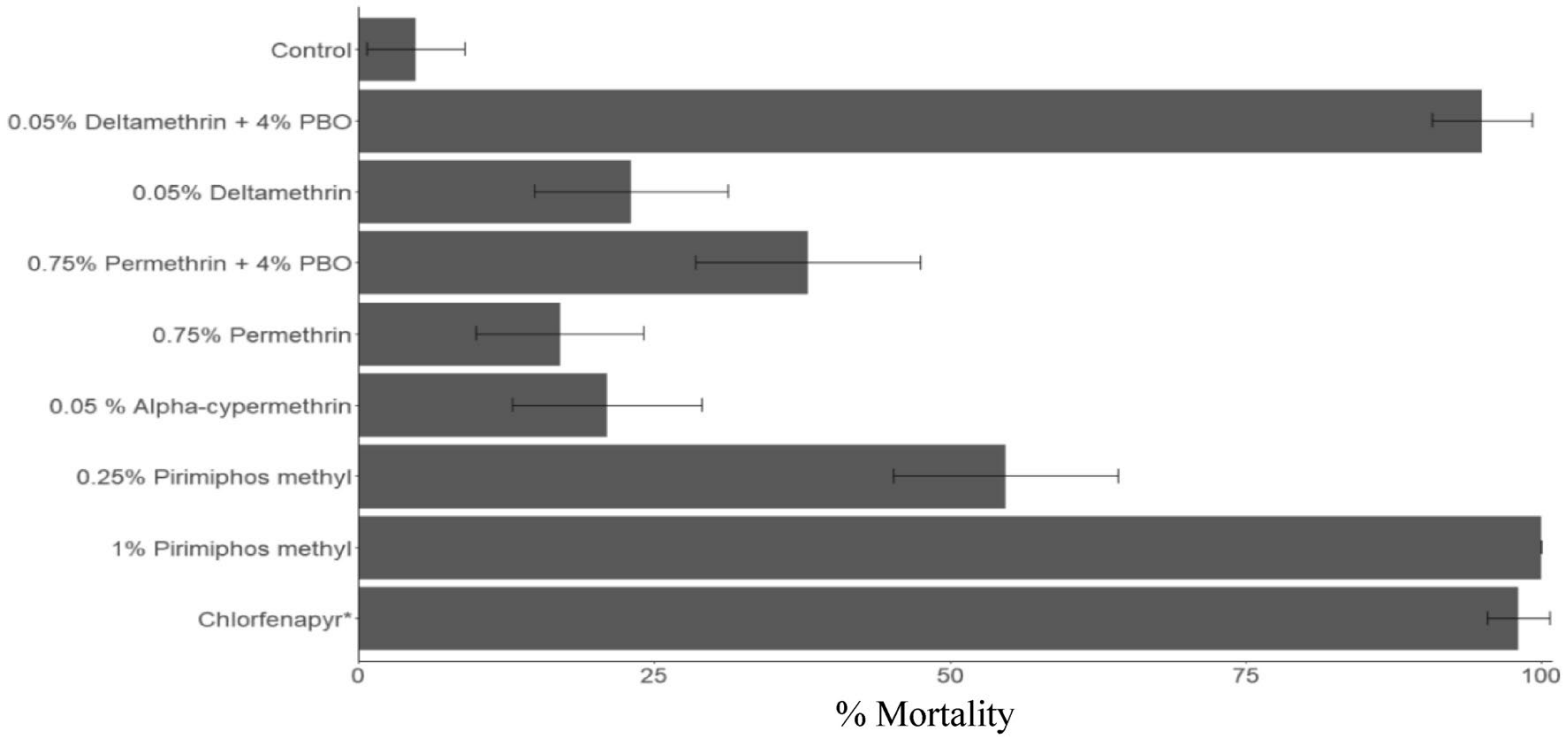
Mortality (%) of wild *An. gambiae* s.l. from Bouaké, Côte d’Ivoire exposed to insecticides in WHO susceptibility bioassays. Error bars indicate 95% confidence intervals. *Susceptibility assays with the pyrrole insecticide chlorfenapyr was performed using CDC bottle assays.

The mortality rates of *An. gambiae* mosquitoes exposed to the discriminating concentrations of the active ingredients in PermaNet 3.0, Interceptor G2, Olyset Plus, and to pirimiphos methyl are presented in Fig. 3. Mortality with the pyrethroid insecticides were less than 25%, indicating a high prevalence of resistance to this class of insecticide. Pre-exposure to PBO resulted in a significant increase in mortality in the pyrethroid resistant *An. gambiae* mosquitoes, from 17 to 38% with permethrin (χ^2^_1_ = 10.69, *P* = 0.001) and from 23 to 95% with deltamethrin (χ^2^_1_ = 107.8, *P* < 0.001). While *An. gambiae* mosquitoes exhibited high resistance to the 0.25% pirimiphos methyl discriminating concentration (54.7% mortality), effective susceptibility was restored (100% mortality) when the dose was increased four-fold to 1%. Chlorfenapyr produced 98% mortality confirming susceptibility to this non-neurotoxic insecticide.

### Semi-field performance of new generation LLINs and pirimiphos methyl treatment deployed as part of a “lethal house lure”

Results from the overnight release-recapture experiments are summarised in Table 1. A total of 4774 female *An. gambiae* mosquitoes were released over the release-recapture study period. The proportion of mosquitoes recaptured was consistently high in all experiments (> 89% mosquito recapture rate).

**Table 1:** Semi-field release-recapture results of insecticidal EaveTube against pyrethroid resistant *Anopheles gambiae* s.l. in enclosure.

	Untreated insert	Beta-cyfluthrin treated insert	PermaNet 3.0 (deltamethrin + PBO)	Olyset Plus (permethrin + PBO)	Interceptor G2 (alphacypermethrin + chlorfenapyr)	Tubes treated with pirimiphos methyl at 10 g/m^2^
Total released	759	811	754	809	796	807
% Recaptured (95% C.I.)	93.5 (91.7–95.2)	91.5 (89.6–93.4)	89.5 (87.2–91.8)	92.6 (90.8–94.4)	94.8 (93.3–96.3)	94 (92.4–95.6)
% Mortality (95% C.I.)	3.52^a^ (2.2–4.9)	62.8^b^ (59.3–66.3)	50.4^c^ (46.6–54.2)	25.9^d^ (22.8–29)	21.6^d^ (18.7–24.5)	66.8^b^ (63.4–70.1)

Values along a row bearing the same letter label are not significantly different (GLMMs, P > 0.05).

Mortality of *An. gambiae* mosquitoes released was significantly higher with all insecticidal tubes (21.6–66.8%), compared to the untreated control tube (< 5%) (*P* < 0.001).

Inserts treated with 10% beta-cyfluthrin killed a greater proportion of pyrethroid resistant *An. gambiae* (62.8%) than any of the new generation nettings (*P* < 0.001). PermaNet 3.0 was the best performing netting, killing about half of the mosquitoes recaptured (50.4%) and the difference in kill rate compared to Olyset Plus (25.9%) and Interceptor G2 (21.6%) was significant (*P* < 0.001). Although mortality with Olyset Plus was higher than that with Interceptor G2, the difference in efficacy was not significant (*P* = 0.35).

Mortality with the 10% pirimiphos methyl treated tube (66.8%) was higher than all of the LLINs (21.6–50.4%), *P* < 0.001) but did not differ significantly from beta-cyfluthrin (62.8%, *P* = 0.57).

### Residual activity

**Figure 4:**
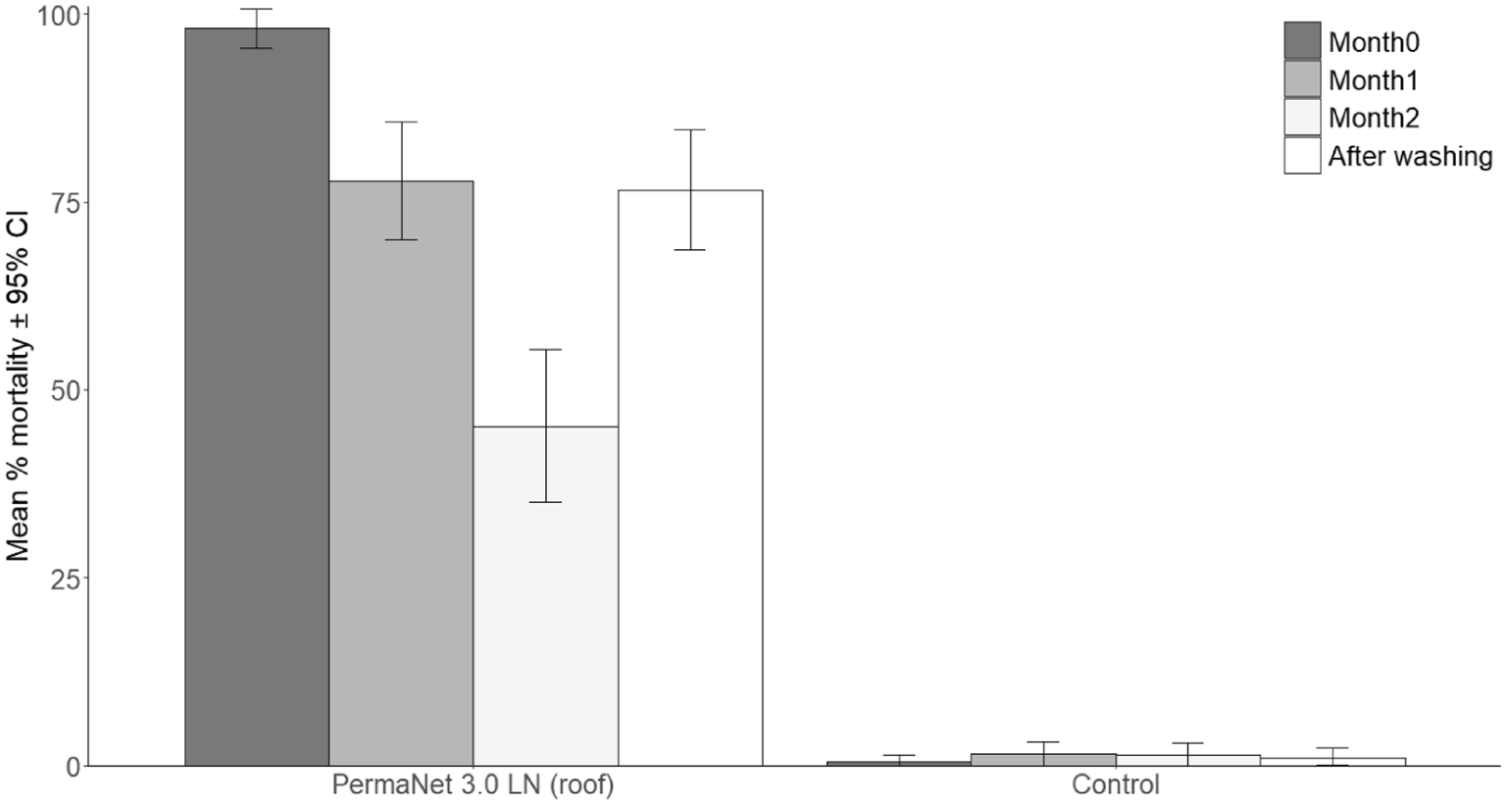
Residual activity in ET bioassays of netting samples from PermaNet 3.0 (roof) LN tested against pyrethroid resistant *Anopheles gambiae* mosquitoes from Bouake with 1 h exposure and 24 h recovery. Error bars indicate the 95% confidence intervals. “After washing” corresponds to Month2 net samples washed 1X.

**Figure 5:**
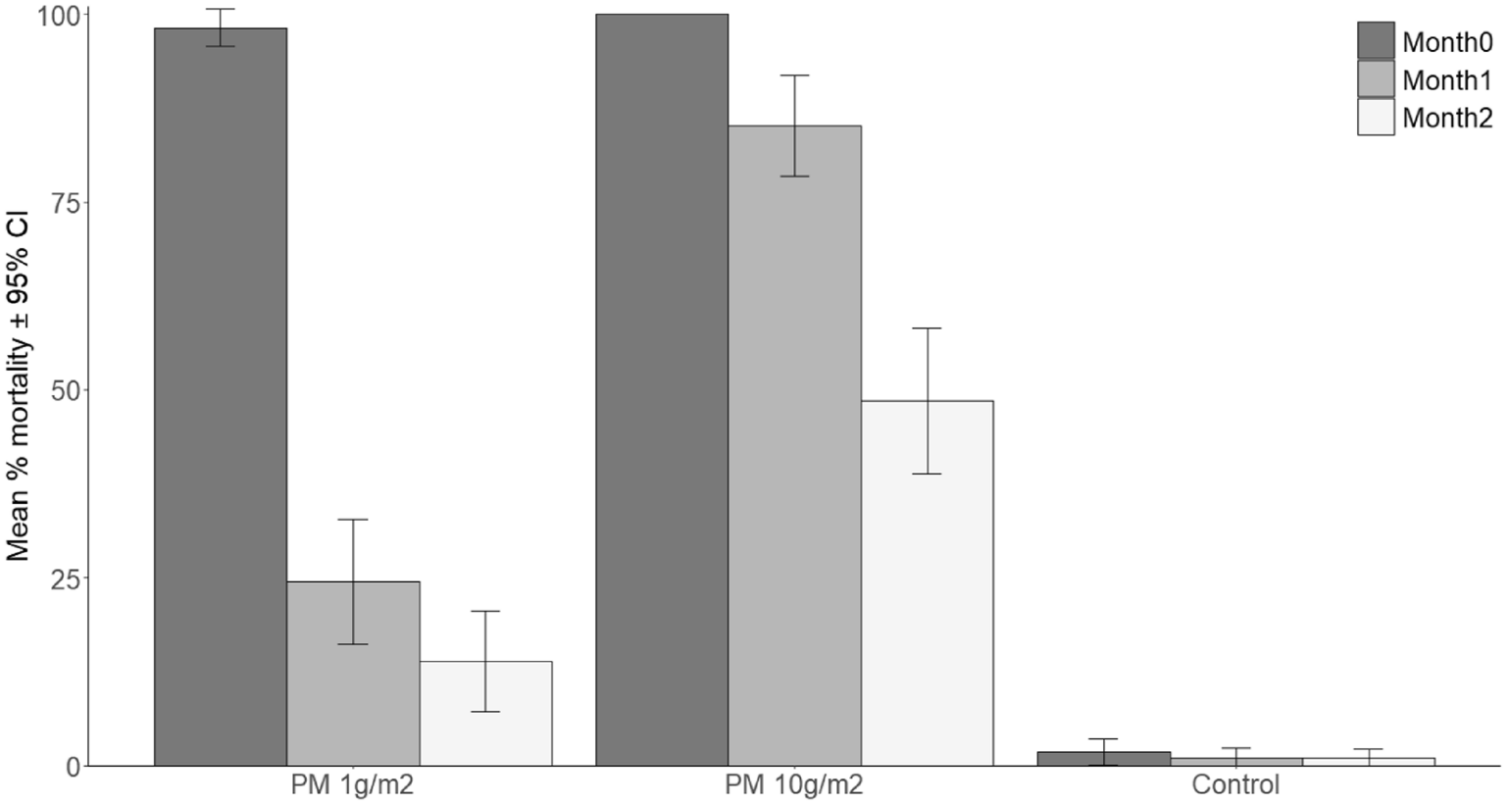
Residual activity in ET bioassays over 2 months of PVC tube coated with pirimiphos methyl at 1 g/m^2^ and 10 g/m^2^ tested against pyrethroid resistant *Anopheles gambiae* mosquitoes from Bouaké with 1 h exposure and 24 h recovery. Error bars indicate the 95% confidence intervals.

Based on results from the release-recapture experiments, only PermaNet 3.0 and pirimiphos methyl coated tubes were assessed further for residual efficacy at different time points (Figs. 4 , 5).

PermaNet 3.0 LN samples in tubes killed a significantly higher proportion of *An. gambiae* mosquitoes compared to the untreated control net (< 5% mortality, Fig. 4). Mortality with fresh PermaNet 3.0 netting was 98.1%; however, efficacy decreased significantly over time, down to 77.8% by month 1 (*P* = 0.005) and 45.2% by month 2 (*P* < 0.001). Washing PermaNet 3.0 after month 2 resulted in a significant increase in mortality compared to the unwashed PermaNet 3.0 at month 2 (from 45.2 to 76.6, *P* < 0.01).

Both doses of pirimiphos methyl (0.25% and 1%) resulted in > 98% mortality in pyrethroid resistant *An. gambiae* at month 0 (*P* = 0.96, Fig. 5). Although the higher dose was still effective at month 1 (> 80% mortality), there was a significant decrease in efficacy by 75% with the lower dose (*P* < 0.01). By month 2, efficacy with the 1% pirimiphos methyl declined by about 50% compared to month 0, but the reduction in activity was much greater with the 0.25% pirimiphos methyl (up to 86%). This indicates a dose-dependent persistence with the higher dose of pirimiphos methyl retaining significantly greater residual efficacy over the 2-month testing period.

### Chemical analysis

The mean deltamethrin and PBO content in the pieces of PermaNet 3.0 nettings are presented in Table 2. The initial concentration of deltamethrin (4.09 g/kg) in PermaNet 3.0 was close to the target dose of 4 g/kg ± 25%. Likewise, the dose of the synergist PBO (24.1 g/kg) in unwashed PermaNet 3.0 was close to the target concentration of 25 g/kg ± 25%. The mean deltamethrin content in the 2-month-old PermaNet 3.0 netting following one wash was 3.5 g/kg, which was still within the target concentration range (3–5 g/kg), although the PBO content was halved (from 24.1 to 11.42 g/kg) (Table 2).

**Table 2:** Content of deltamethrin and piperonyl butoxide (PBO) in the roof panel of PermaNet 3.0 LN used in release-recapture experiments.

Treatment	Concentration of deltamethrin (g/kg)	Concentration of PBO (g/kg)
Unwashed PermaNet 3.0 LN (roof)	4.09	24.1
2-month-old PermaNet 3.0 LN (roof) washed 1 X	3.5	11.42

## Discussion

With the international effort to identify new approaches for controlling malaria, there is increasing interest in house modification that could lead to reduced risk of malaria transmission. The In2Care EaveTube is an example of such an intervention. It is designed to block mosquito entry points and kill mosquitoes as they attempt to enter the house, by insertion of insecticide-treated electrostatic netting in their path to the interior of the house via the eave gap. The present study builds on previous work on the resistance breaking potential of netting electrostatically treated with insecticide powders under laboratory and semi-field conditions. The aim of the current study was to 1) evaluate the residual efficacy of beta-cyfluthrin treated inserts placed in inhabited village houses as part of the CRT, and to 2) further explore alternative technologies for delivering insecticides in tubes using a combination of laboratory and semi-field experiments.

The bioefficacy and residual activity of beta-cyfluthrin on inserts deployed in trial villages showed mosquito mortality below 80% four months after treatment during the first two rounds despite higher impact (> 80%) in subsequent rounds. Although freshly treated inserts were bio-effective against pyrethroid resistant mosquitoes, the residual activity recorded in the present study was much shorter than in a previous study which showed > 80% mortality for over 9 months^16^. This disparity could be due to differences in insecticide application method; inserts deployed in the trial villages were treated using an ‘insecticide application machine’^39^ developed by In2Care, while in the previous study, inserts were treated by hand^16^. It is possible that the amount of insecticide deposited by machine treatment was lower than that deposited by hand treatment. This seems likely since the first application round was particularly poor and the application apparatus was subsequently modified to deliver more powder with more uniform coverage, and this appears to be reflected in improved persistence. The short residual efficacy of beta-cyfluthrin treated insert reported in the CRT suggests that high frequency of insecticide re-treatment will be required in year-round malaria transmission settings. To address the poor residual efficacy, a couple of residual efficacy studies have recently been conducted to screen and identify products with longer duration of effective action. Deltamethrin powder applied at a dosage of 5% was shown to provide long lasting control of both susceptible (100% mortality over 18 months)^46^ and resistant *Anopheles* mosquitoes (> 95% mortality over 12 months)^47^ under village conditions of use.

While female mosquitoes of endophilic malaria vectors rest on insecticide-treated house walls long enough to pick up a lethal dose of insecticide even when slow-acting chemistries are deployed^34,48^, evidence from filming studies show that mosquitoes attempting to enter people’s dwellings via eave gaps in search for a blood meal spend on average < 5 min on insecticide-treated inserts^49^. This suggests that, in order to be effective, the insecticide in the tube should have the attributes of fast-killing and high toxicity with capacity to control insecticide resistant mosquitoes with an exposure time of just a few minutes. The current insecticide delivery system used in the EaveTube strategy—the electrostatic coating—meets these criteria and was shown to bypass resistance even under scenario of transient contact time through enhanced bioavailability and high transfer of insecticide^20^. Although the electrostatic coating has demonstrative potential, the development of new insecticides and new formulations provides opportunities for alternative insecticide delivery methods in the lethal house lure. The semi-field performance of nettings from new generation LLINs and tubes coated with pirimiphos methyl was evaluated in experimental huts and compared to 10% beta-cyfluthrin treated insert. The kill rate with beta-cyfluthrin (63%) was in the same range as the mortality rates produced by tubes treated with pirimiphos methyl (66.8%). The mortality observed was broadly consistent with results from previous studies of insecticide treated EaveTubes conducted at the same study site and in East Africa^12,13,16,17^. It is worth noting that the ~ 50% mortality induced by these treatments corresponds to the actual proportion of female mosquitoes contacting the tube over a release-recapture study night (~ 44%)^16^.

The level of efficacy achieved with the top side of PermaNet 3.0 netting and tube treated with pirimiphos methyl (> 50% mortality) in release-recapture experiments is predicted to have significant impact on malaria transmission according to a recent mathematical modelling study^50^. This suggests that alternative mode of delivery of insecticides including pieces of netting from synergist LLINs, and eave tube dipped in insecticide solution (pirimiphos methyl) could be used in “Lethal House Lure” approach for malaria control.

Although all the fresh new generation LLINs tested were efficacious against pyrethroid resistant female mosquitoes in the semi-field trial, the magnitude of the impact was significantly lower with Olyset Plus (permethrin and PBO) and Interceptor G2 (alpha-cypermethrin and chlorfenapyr) than with PermaNet 3.0 (deltamethrin and PBO). The difference between the roof of PermaNet 3.0 and Olyset Plus LLINs is likely due to the difference in the levels of toxicity of the pyrethroids in the nets. PermaNet 3.0 is impregnated with type II pyrethroid deltamethrin, whereas Olyset Plus is treated with type I pyrethroid permethrin. There is evidence that type II pyrethroids, which contains an alpha cyano group, are more toxic than type I pyrethroids^51^. This is supported by the results of the WHO susceptibility assays with deltamethrin killing significantly higher proportion (95%) of pyrethroid resistant mosquitoes pre-exposed to PBO compared to permethrin (38%). In addition to the difference in the type of pyrethroid used in these nets, the dose of PBO in the roof of PermaNet 3.0 (25 g/kg) is almost three times higher than that in Olyset Plus LN (10 g/kg).

The performance of the dual-active Interceptor G2 was unexpected given prior evidence from experimental hut studies with human occupied IG2 LN demonstrating high efficacy against wild free-flying pyrethroid resistant mosquitoes^32,52^. Susceptibility to chlorfenapyr was confirmed in CDC bottle bioassays. However the efficacy of this non-neurotoxic insecticide depends on a number of factors including exposure duration and the mosquito’s circadian activity^30^. Chlorfenapyr is a pro-insecticide which is converted by P450 enzymes into its potent form at night, when mosquitoes are active. Because the release-recapture studies were conducted overnight, it is unlikely that the low mortality observed was a result of chlorfenapyr not being metabolised to its toxic form. On the other hand, given that the interaction between host-seeking mosquitoes and tubes is relatively transient in EaveTubes^49,53^, it is possible that the exposure duration on the mixture net was not sufficiently long for the mosquitoes to pick up a lethal dose of chlorfenapyr which could account for the low mortality induced by Interceptor G2.

The residual efficacy of the alternatives in the tubes was low, and none of the products showed effective control of pyrethroid resistant mosquitoes beyond 2 months. Pirimiphos methyl was short-lived, even when a higher concentration was used. The low persistence of Actellic CS reported in the present study contrast with results from previous experimental huts and randomized controlled trials demonstrating much longer residual activity of pirimiphos methyl (≥ 75% mortality for ~ 1 year) on wall substrates commonly found in rural African houses^33,54^. The low persistence was potentially due to the difference in substrate type (cement wall versus plastic tube). It could also be that dust accumulation and environmental factors such as humidity, temperature and UV exposure might have contributed to the rapid decline in activity on the treated tubes^55^. Treating the inserts themselves, which are on the inside of the tubes in line with the inner side of the house wall, could potentially reduce exposure to some of these elements. In addition, regardless of current challenges for persistence, it is noteworthy that the mortality from the pirimiphos methyl resulted from treatment of the tube rather than the insert. These results suggest it might be possible to increase the impact of EaveTubes as treating both the tubes and the insert could increase the total kill zone.

The residual activity of active ingredients in the new generation LN, PermaNet 3.0 (roof), was also short with mortality rates decreasing below 50% within 2 months. Since the nettings were directly exposed to environmental conditions, it is likely that the same factors mentioned above might have combined to degrade the insecticide in the nets. Washing PermaNet 3.0 roof resulted in a partial recovery in efficacy, which was consistent with the chemical analysis results. Indeed, about half the initial concentration of PBO remained in the 2-month-old PermaNet 3.0 netting after one wash, which appeared sufficient to neutralize metabolic enzymes and restore net efficacy to some extent. Nevertheless, the rapid decline in PBO content could impact persistence in the eaves.

The nets tested in the present study are treated with established concentration of insecticides based on use. However, since nets are deployed in tubes that are placed at eave height, and therefore out of reach of house residents, higher than currently recommended doses of insecticides in nets and chemistries not allowed on net due to safety concerns could be considered to improve efficacy and duration of effective action. Likewise, based on the dose-dependent efficacy and persistence pattern with pirimiphos methyl and the position of tubes at eave level, tube could be treated with higher concentrations of insecticides to provide prolonged control of insecticide resistant mosquitoes while minimising exposure to house occupants.

## Conclusion

Beta-cyfluthrin powder showed lower persistence on treated In2Care EaveTube inserts used in the CRT than suggested in earlier studies, possibly due to changes in powder application methods. In the first round of application in the CRT, persistence was less than 2 months. Modifications to the powder application technology increased this to around 4 months in later treatment rounds. It is likely that improvements in formulation, application methods and possibly use of different actives, could potentially increase persistence further. In addition, other types of delivery methods (and associated actives), could also open up opportunities for improving persistence and create new possibilities for resistance management. The current study provided proof of principle that LLIN- and IRS-type treatments could be used to deliver insecticides within an EaveTube. However, none of these products appeared superior to the powder treatments. Overall, this research points to the need for further product development to explore the potential of this promising control tool.

## Data Availability

All data generated or analysed during this study are included in this published article.
